# Bayesian Optimization in Bioprocess Engineering—Where Do We Stand Today?

**DOI:** 10.1002/bit.28960

**Published:** 2025-03-05

**Authors:** Florian Gisperg, Robert Klausser, Mohamed Elshazly, Julian Kopp, Eva Přáda Brichtová, Oliver Spadiut

**Affiliations:** ^1^ Christian Doppler Laboratory for Inclusion Body Processing 4.0 Vienna Austria; ^2^ Research Area Biochemical Engineering, Institute of Chemical, Environmental and Bioscience Engineering Technische Universität Wien Vienna Austria

**Keywords:** active learning, Bayesian optimization, bioprocess engineering, machine learning, model‐based optimization

## Abstract

Bayesian optimization is a stochastic, global black‐box optimization algorithm. By combining Machine Learning with decision‐making, the algorithm can optimally utilize information gained during experimentation to plan further experiments—while balancing exploration and exploitation. Although Design of Experiments has traditionally been the preferred method for optimizing bioprocesses, AI‐driven tools have recently drawn increasing attention to Bayesian optimization within bioprocess engineering. This review presents the principles and methodologies of Bayesian optimization and focuses on its application to various stages of bioprocess engineering in upstream and downstream processing.

## 1 Introduction

In science and engineering, progress and uncovering new knowledge depend on systematic experimental investigation of complex systems. Especially in biological systems, where the prediction accuracy of experimental outcomes is highly data‐dependent, the approach to experimentation has evolved significantly over time. The most straightforward systematic experimental design is the One‐Factor‐At‐a‐Time (OFAT) approach, which involves altering a single variable within a system while keeping the others constant and observing the system's response to each alteration (Daniel 1973). However, while being straightforward and intuitive, by design, the effect of only one variable on the system is observed (Razavi and Gupta 2015). Furthermore, the limitations of manually executable experiments are quickly reached in multifactorial systems, as the number of total experiments increases exponentially with the number of parameters to be screened. This led to the statistical Design of Experiments (DoE) method, which constitutes a significant improvement introduced by Ronald Fisher in 1935 (Fisher 1935) and further refined by George Box in 1951 (Box and Wilson 1951). By introducing response surface models, DoE became a new experimental design and optimization standard.

In general, a response surface model is an empirical representation of the effect of several input variables on a target response fit using regression techniques based on experimental data. The choice between linear, polynomial, or other model forms depends on the complexity of the process studied. This choice directly influences the sampling strategy, ranging from full factorial designs for simple systems to fractional factorial or central composite designs for more complex interactions. Full factorial designs cover all factor combinations but are often impractical for studies with many variables, leading to the use of fractional designs that sample a subset of combinations to identify main effects and interactions efficiently. Central composite designs further refine the approach for quadratic models, optimizing the exploration of nonlinear relationships (Szpisják‐Gulyá's 2023).

However, a limitation of DoE is that it requires a predetermined mathematical model, such as a linear or polynomial, from the start. It creates a bias that may not accurately reflect the underlying system dynamics (Razavi and Gupta 2015). Furthermore, traditional DoE methods tightly integrate sampling and result analysis, limiting the flexibility to adapt to new findings during the experimental process. This approach can limit responsiveness to new findings, hindering immediate adjustments based on experimental outcomes (Greenhill et al. 2020). Iterative extensions of classical DoE methods, such as sequential optimal designs address these issues by incorporating new data into the design process (Goos 2006; Wynn 1970). These approaches improve model accuracy and efficiency in identifying optimal solutions. However, complementing both traditional and iterative DoE methods, adaptive experimentation methodologies provide a broader framework that evolves based on real‐time data and insights. Adaptive methodologies often go beyond refining models to explicitly guide exploration and exploitation in experimental designs. This adaptive approach enables scientists to adjust to new findings during experiments. The following samples can be taken from previously unexplored areas that are likely to yield maximum or minimum values or represent a compromise. Adaptive sampling methods could lead to finding the optimal solution more quickly while reducing the number of experiments (Jin and Kumar 2023).

In recent years, Bayesian optimization (BO) has emerged as a logical progression to traditional DoE methods. Initially postulated by Jonas Mockus in 1975 (Močkus 1975) and gaining prominence in the 2010s for optimizing neural network hyperparameters (Snoek et al. 2012), BO integrates Machine Learning with adaptive sampling into a flexible, model‐based global optimization algorithm. BO, like DoE, relies on an underlying mathematical model. Often, this model takes the form of Gaussian processes (GP), which are highly flexible, non‐parametric models capable of representing complex, nonlinear relationships without requiring a predefined functional form. They are very effective with small datasets and stochastic noise, while their ability to quantify uncertainty enables BO to dynamically balance exploration and exploitation during optimization. This makes BO efficient in identifying optimal solutions with minimal experimental effort (Frazier 2018).

BO's versatility and efficacy in addressing complex optimization challenges have been demonstrated across various fields, including materials science (Jin and Kumar 2023; Zhang 2021; Rohr et al. 2020; Chepiga 2023; Frazier and Wang 2015), chemical reactions (Pickles 2024; Zhang 2024), protein engineering (Hu et al. 2023), neuroscience (Choinière 2024) and improving cookie recipes (Daniel Golovin 2017), among others.

However, to date the application of BO in bioprocess engineering remains relatively underexplored compared to traditional Design of Experiments methods (Kasemiire 2021). This review article first outlines the concept of BO, providing a comprehensive overview of its principles and methodologies. Then, we provide a detailed review of the current literature on the application of BO to problems in bioprocess engineering.

## 2 Bayesian Optimization: A Sequential Model‐Based Approach

BO is an advanced global optimization framework combining a probabilistic surrogate function and an acquisition function. The surrogate function estimates the objective function's behavior using existing data, turning the objective function into an easier‐to‐evaluate surrogate. Conversely, the acquisition function strategically determines new sampling points, balancing the exploration of new areas against the exploitation of known high‐value regions guided by the surrogate model.

The central idea of BO lies within Bayes' theorem (Bayes 1763), which updates the surrogate model iteratively with new data. Bayes' theorem is expressed as

(1) $$P\left(A|B\right)=\frac{P\left(B|A\right)P\left(A\right)}{P\left(B\right)}$$

where *P*(*A* | *B*) is the posterior probability of the parameters *A* given the data *B*, *P*(*B* | *A*) is the likelihood of the data B given the parameters A, *P*(*A*) is the prior probability of the parameters, and *P*(*B*) is the probability of the data.

**Figure 1:**
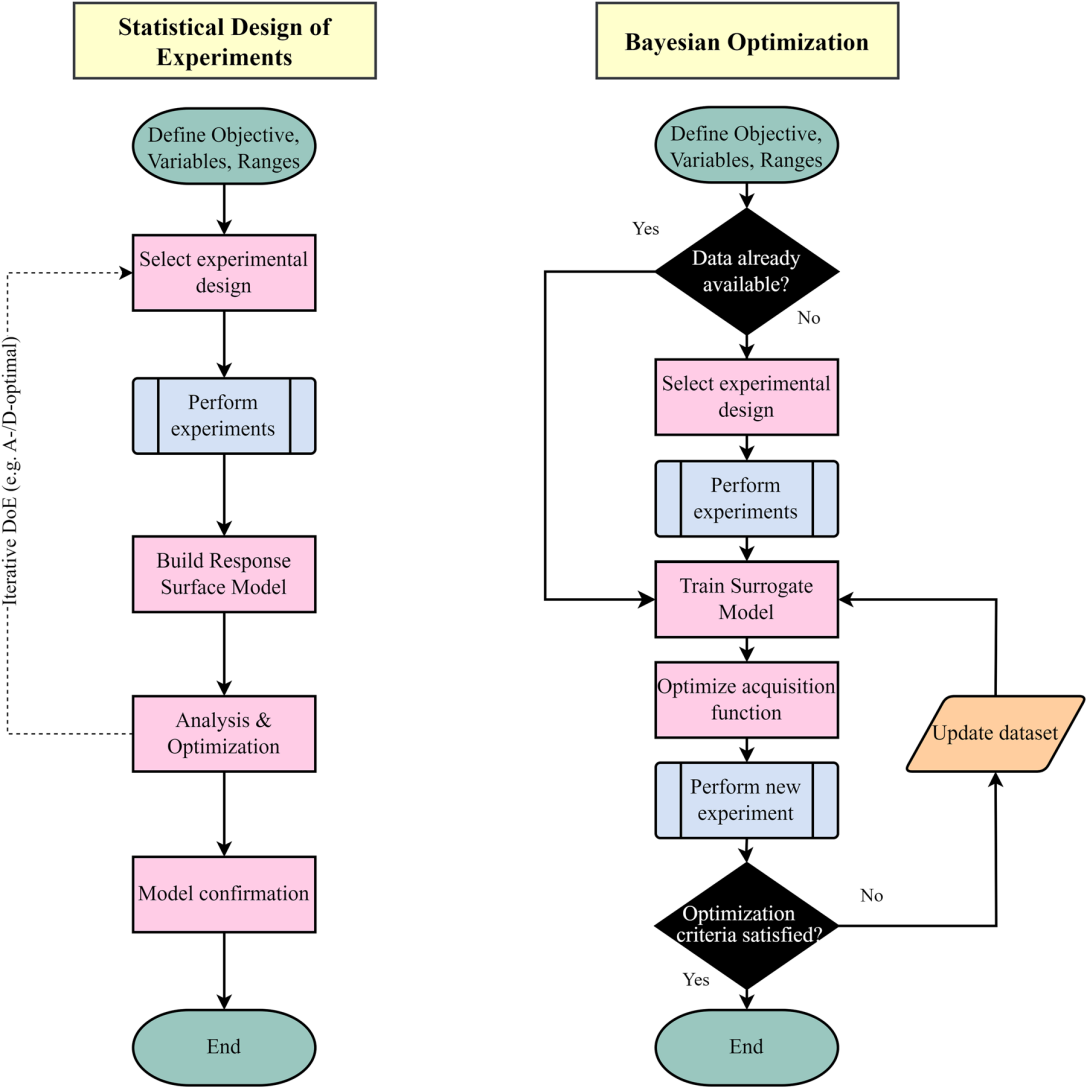
Comparing the sequence of DOE versus BO: Statistical design of experiments typically follows a structured approach, often using models like Response Surface Methodology to analyze relationships between variables. DOE can include iterative cycles, enabling refinement of experimental designs based on initial results. BO, by contrast, adopts an inherently adaptive strategy, using a probabilistic surrogate model and acquisition function to dynamically guide experiments.

This theorem mathematically demonstrates how existing knowledge is updated, allowing for a dynamic and iterative learning process. This approach diverges from the traditional DoE by considering uncertainty and incorporating a decision‐making policy. Both workflows are presented in Figure 1.

In the following paragraphs, surrogate functions and acquisition functions are introduced.

### 2.1 Surrogate Function

BO uses a surrogate model to approximate the unknown black‐box function *f*, with the GP model being the most popular due to its natural element of uncertainty quantification and its flexibility (Rasmussen and Williams 2006). The GP surrogate model is defined by a mean function *m*(*x*) and a covariance function, or kernel, *k*(*x,x* ^′^), articulated as

(2) $$f\left(x\right)∼\text{GP}\left(m\left(x\right),k\left(x,x^{\prime }\right)\right)$$

The mean function *m*(*x*) represents the average output of the function *f*(*x*) across the design space, often assumed to be zero for simplicity in many applications.

The kernel function *k*(*x,x* ^′^) defines the covariance between any two points *x* and *x* ^′^ in the input space, capturing a measure of similarity between them. This similarity determines how relationships in the data are modeled, impacting the GP's ability to represent smoothness and variability in *f*(*x*) (Garnett 2023). Kernels are particularly powerful because they implicitly map inputs into a high‐dimensional (or even infinite‐dimensional) feature space without explicitly performing the transformation. This is achieved through the kernel trick, where the kernel function directly computes inner products in the feature space. In this way, complex, nonlinear patterns in the input space can be represented as simpler, linear relationships in the implicit feature space, enabling GPs to model a wide range of functional behaviors without requiring explicit parameterization of *f*(*x*) (Rasmussen and Williams 2006).

The choice of kernel governs the GP's flexibility and adaptability, with different kernels encoding assumptions about smoothness, variability, or periodicity (Rasmussen and Williams 2006). For example, a commonly used kernel is the squared exponential (SE) function, also known as the radial basis function (RBF), defined as

(3) $$k\left(x,x^{\prime }\right)=\text{exp}\left(-\frac{1}{2\theta^{2}}{\left|x-x^{\prime }\right|}^{2}\right)$$

where *θ* is the length scale parameter controlling the smoothness of the function. Another important class of kernels is the Matérn kernels, a generalization of the RBF kernel, providing additional flexibility through the smoothness parameter *ν (*Rasmussen and Williams 2006 *)*. This parameter controls the differentiability of the modeled function and a particularly useful variant of the Matérn kernel occurs when *ν* = 5*/*2. This specific case offers a good balance between smoothness and computational traceability and is also commonly used besides RBF (Snoek et al. 2012).

While GPs are highly flexible and effective, their computational cost scales cubically (O(*n* ^3^)) with the number of data points *n*. This is because GPs involve the inversion of an *n* × *n* covariance matrix during training, making them less suitable for very large datasets. Approximations such as sparse GPs or inducing point methods can mitigate this issue, but trade‐offs in accuracy may occur (Csató and Opper 2002; Wang and Chen 2023).

As BO relies on a model's ability to quantify uncertainty for efficiently searching for optimal solutions, every model offering this ability could be used as a surrogate. Although GPs are often the preferred choice due to their inherent probabilistic nature, other models such as Random forests (Qi et al. 2024) and Bayesian neural networks (Snoek 2015) can be adapted for BO. Additionally, deep ensemble methods can provide uncertainty estimates by analyzing the variance in predictions from multiple models. Even non‐probabilistic models can be integrated into BO using surrogate methods or prediction errors to approximate uncertainty (Westermann and Evins 2020).

It is worth noting that the reliability of uncertainty quantification depends heavily on the choice of hyperparameters, priors, and model structures. While optimization routines like marginal likelihood maximization reduce subjectivity, these choices still rely on domain knowledge and assumptions, which can influence the results (Snoek et al. 2012).

### 2.2 Acquisition Function

**Figure 2:**
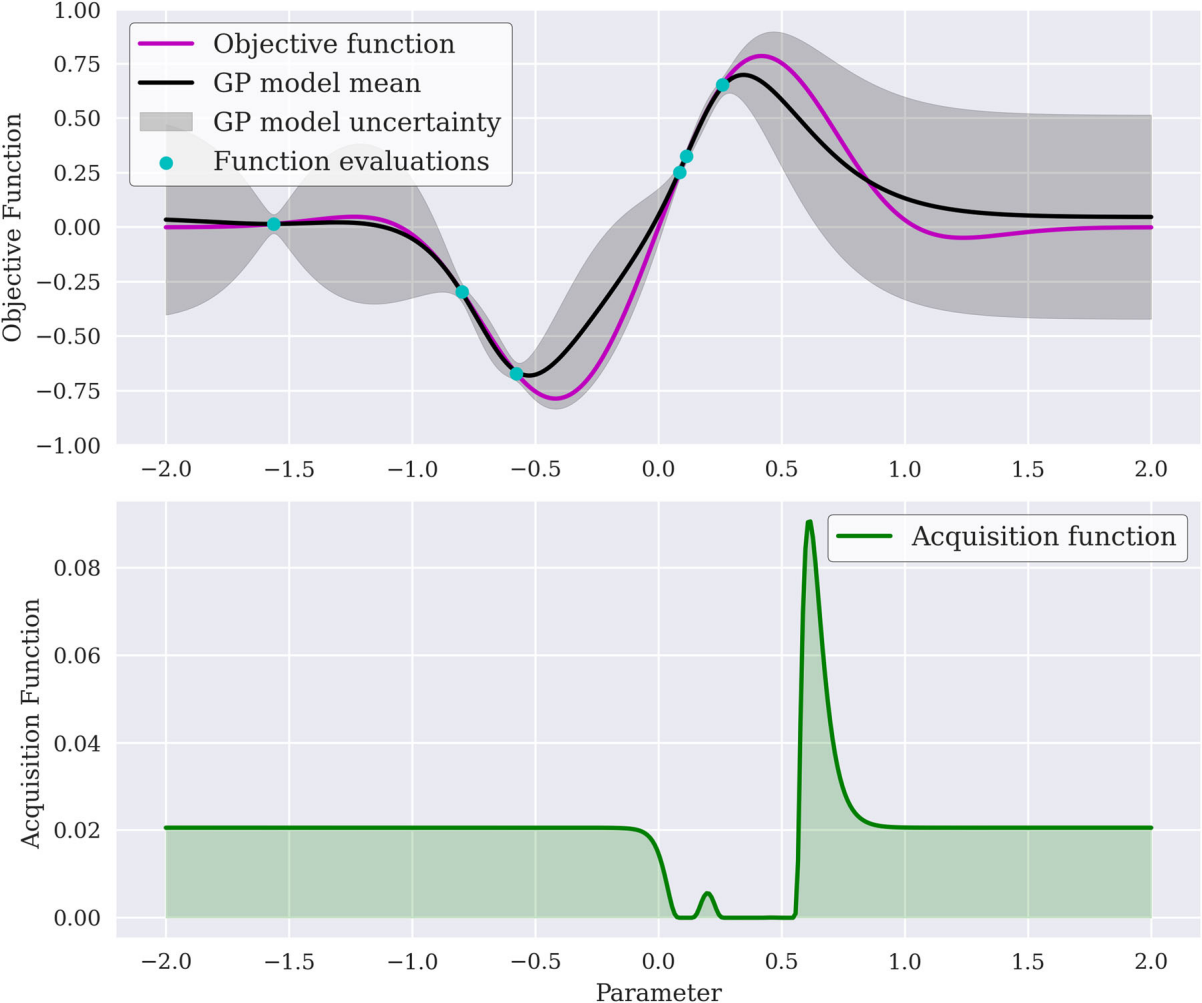
Demonstration of the interplay between Gaussian process regression and acquisition function within a BO loop. Depicted is the Expected Improvement acquisition function indicating the next sampling point at its peak, balancing exploration versus exploitation.

As the decision‐making strategy, acquisition functions in BO determine the next point to query in the optimization process. They utilize the mean *µ*(*x*) and uncertainty *σ*(*x*) predictions from the GP model as a basis for optimization. A balance is typically sought between exploiting areas with a known high objective mean and exploring regions with significant uncertainty (Greenhill et al. 2020). The principle is illustrated in Figure 2. Based on this trade‐off, acquisition functions are classified into three primary categories: improvement‐based, optimistic, and information‐based (Jin and Kumar 2023).

Improvement‐based policies prioritize candidates expected to improve upon the current optimum. The Probability of Improvement (PI) is an example and is formulated as

(4) $$\mathrm{PI}\left(x\right)=\Phi \left(\frac{\mu_{t}\left(x\right)-f\left(x_{t}^{+}\right)-\xi }{\sigma \left(x\right)}\right)$$

where *µ*_*t*_(*x*) represents the predicted mean of the objective function at point *x*, *f*(*x* ^+^_*t*_) is the current maximum observed value of the objective function, *ξ* is a small positive number to encourage exploration by prioritizing regions with higher uncertainty, *σ*(*x*) indicates the model's predicted standard deviation or uncertainty at point *x* and Φ is the cumulative distribution function of the standard normal distribution. This balances the exploration of unknown regions with the exploitation of known promising areas (Kushner 1964).

The expected improvement (EI) is another improvement‐based acquisition function that considers both the likelihood and the expected magnitude of improvement over the current best observation. It is defined as

(5) $$\mathrm{EI}\left(x\right)=\left(\mu_{t}\left(x\right)-f\left(x_{t}^{+}\right)\right)\Phi \left(Z\right)+\sigma_{t}\left(x\right)\phi \left(Z\right)$$

where

(6) $$Z=\frac{\mu_{t}\left(x\right)-f\left(x_{t}^{+}\right)}{\sigma_{t}\left(x\right)}$$

represents the standardized distance between the predicted mean *µ*_*t*_(*x*) at point *x* and the current maximum observed value *f*(*x* ^+^_*t*_), scaled by the predicted standard deviation *σ*_*t*_(*x*) at point *x*. This standardization transforms the difference into a dimensionless quantity, allowing it to be used directly in the calculations of Φ and *ϕ*, where Φ is the cumulative distribution function and *ϕ* is the probability density function of the standard normal distribution (Močkus 1975).

Optimistic policies such as the upper confidence bound (UCB) favor exploration by assuming regions of high uncertainty can yield better outcomes:

(7) $$\mathrm{UCB}\left(x\right)=\mu_{t}\left(x\right)+\kappa_{t}\sigma_{t}\left(x\right)$$

where *κ* is a tuning parameter that balances the level of exploration against exploitation (Srinivas et al. 2012).

Information‐based policies, like entropy search (ES) and knowledge gradient (KG), are designed to maximize the information gained about the optimum's location or the expected improvement in the surrogate model's maximum (Frazier et al. 2008).

Custom acquisition functions are particularly relevant for scenarios where experiments are conducted in batches. These functions, such as batch expected improvement (qEI) and batch upper confidence bound (qUCB), determine multiple potential candidates at once using Monte Carlo sampling and reparameterization techniques for efficient selection (Balandat 2020).

### 2.3 Advanced Methods in Bayesian Optimization

The core iterative process of BO has been adapted and extended to meet challenges presented by various real‐world problems. The most important principles are presented in this section. However, in‐depth explanations can be found in the following reviews (Greenhill et al. 2020; Jin and Kumar 2023; Shahriari 2016; Wang 2023).

**Incorporating Prior Knowledge.** Leveraging prior knowledge in the BO framework can significantly enhance optimization efficiency by reducing search complexity. As Bayes' theorem fundamentally is about updating knowledge, e.g., similar data from past experiments can be used as a prior and leveraged in different ways (Joy 2019). Furthermore, the Gaussian process model as such offers mechanisms to insert expert knowledge, for example, by editing the kernel or the mean function itself (Nguyen et al. 2023).

**Mixed‐Type Space.** Many optimization problems involve mixed types of input variables (continuous, discrete, categorical, and binary). BO has been adapted to handle such mixed‐type inputs through methods, like one‐hot encoding, kernel modification to accommodate different types of variables, or using surrogate models like Random forests that naturally handle mixed inputs (Shields et al. 2021).

**High Dimensionality.** Generally, the performance of BO is decreasing above 20 dimensions (Greenhill et al. 2020). Strategies to mitigate this include reducing the design space, screening variables to identify the most influential ones, decomposing the problem into simpler sub‐problems, or employing dimensionality reduction techniques. Specialized kernels and approximation methods have been developed to enable BO in higher‐dimensional spaces.

**Multi‐Objective Optimization.** Design problems often encompass multiple conflicting objectives. BO has been adapted to handle such scenarios through the concept of Pareto optimization, where a set of optimal points is identified so that no single objective can be improved without detriment to others. This approach requires balancing the trade‐offs between objectives and often involves constructing a Pareto front to guide the optimization process (Bradford et al. 2018).

**Constraints.** Real‐world optimization problems frequently come with various constraints, including inequality and equality constraints, as well as “black box” constraints whose forms are unknown. BO methods have evolved to incorporate such constraints into the optimization process, either by modifying the acquisition function or employing strategies like the Augmented Lagrangian method to transform constrained optimization problems into unconstrained ones (Picheny 2016; Letham et al. 2019).

**Parallel (Batch) Optimization.** In scenarios where multiple experiments can be conducted simultaneously, BO has been extended to recommend batches of experimental settings. This approach involves selecting multiple points to evaluate in parallel, using techniques such as suppression or local penalization in the acquisition function to account for the simultaneous exploration of multiple points (Gonzalez 2015).

**Multi‐Fidelity (Multi‐information) Optimization.** When high‐fidelity evaluations are expensive, BO can utilize multi‐fidelity models that combine high‐‐fidelity experimental results with lower‐fidelity information. That might be simulations, predictions, or cheaper but noisier measurements. This approach enables an efficient search by leveraging convenient, lower‐fidelity evaluations to guide exploration and exploiting the more expensive, higher‐fidelity evaluations (Cosenza et al. 2022).

## 3 Application Examples of BO in Bioprocess Engineering

An overview of several studies on BO application in bioprocess engineering published in the past 7 years is given in this section. The selected studies are organized by their respective unit operation.

### 3.1 Upstream Processing

#### 3.1.1 Media Optimization

Yoshida et al. optimized a synthetic medium for plasmid‐based protein expression in *Escherichia coli* using 31 different media components (Yoshida et al. 2023). By a Latin Hypercube experimental design, 81 cultivations were performed in deep‐well‐scale, and GFP fluorescence was measured as a response to protein expression levels. The resulting data set, which consisted of media component concentrations and fluorescence intensities, was used to train a deep neural network (DNN) and showed promising results after a second retraining. This DNN was used as a basis to be optimized by BO, with the algorithm suggesting 20 new media compositions. By this approach, the authors transformed the problem into a digital representation, focusing on optimizing a simulation of the problem instead of engaging in a continuous cycle of laboratory experiments and reassessments. The final GFP fluorescence showed a 1.4‐fold higher intensity than the best of the initial data set and was validated on a larger scale.

In their approach to optimize cell culture media for cellular agriculture, Consenza et al. encountered limitations in prior research determining multi‐passage growth (Cosenza et al. 2021). Therefore, the researchers utilized a multi‐information source BO algorithm in a novel approach. This method combined low‐fidelity assays, like AlamarBlue and LIVE/DEAD staining, which offer cost‐effective biomass estimates, with high‐fidelity measurements such as trypan blue exclusion cell counting (Cosenza et al. 2022). The algorithm aimed to maximize cell growth while minimizing media cost. The optimization of 14 media components for murine C2C12 cells led to a media formulation that significantly outperformed a common commercial medium in supporting cell proliferation, achieving an 181% increase in cell counts with 38% fewer experiments compared to a traditional DoE method. This medium also demonstrated robust performance over long‐term growth across multiple cell passages.

Similarly, as an extension to their multi‐information BO, Cosenza et al. also utilized a multiple‐objective multi‐information BO to develop a serum‐free medium tested with murine C2C12 cells for a cellular agriculture application (Cosenza 2023). The objective being the maximization of cell growth while achieving minimization of cost, the authors discovered media with double the growth than the control.

#### 3.1.2 Cell Culture

Claes et al. explored the efficacy of BO for bioprocess optimization, focusing specifically on its application in cell therapy manufacturing under conditions of deliberately varying experimental noise and the need for parallel experimentation (Claes et al. 2024). The research assessed BO's performance through evaluations on two in silico bioprocess models and further demonstrated its application in an in vitro monocyte purification. The first in silico analysis revealed that BO outperformed the current industry standard DoE, requiring roughly 50% fewer experiments to achieve similar optimization objectives. Specifically, the BO methodology was shown to reduce the experimental load by approximately 69% for the monoclonal antibody (mAb) process optimization, where it achieved a comparable product yield with only 14 experiments, as opposed to the 45 experiments required by traditional response surface methodology. For the in vitro application, the optimization of a monocyte purification process via counterflow cell centrifugation was optimized using BO. This practical application increased monocyte recovery from 46% to 82% while maintaining a purity level of 78%, all within a framework of 10 experimental runs. This application showcased BO's capability to navigate complex bioprocess optimization landscapes efficiently and demonstrated its potential to significantly reduce resource consumption and accelerate development timelines in cell therapy manufacturing.

Bader et al. applied BO for optimizing the production of extracellular vesicles from a 3D culture of mesenchymal stem cells (Bader 2023). Four parameters were considered in the process optimization, namely microcarrier concentration, seeding density, centrifugation time, and impeller speed, to optimize three objectives: maximizing enzymatic activity of the MSG‐EV protein CD73, maximizing vesicle to protein ratio and minimizing the number of calregulin impurities. This was achieved using a multi‐objective batch BO algorithm in 32 (4 × 8) experiments. The first set of eight experiments was planned using a Latin Hypercube design and was used for initial Gaussian process modeling. Comparable DoE designs lie within 27–81 experiments for different experimental designs. The authors also noted that the data set is rich in information due to systematic sampling in regions with high uncertainty, as performed by the acquisition function. It is shown that this data can be used further to build explanatory models for authorities in a quality‐by‐design fashion or for creating predictive models, like an artificial neural network with an error near the analytical error.

De Luca et al. conducted an in silico study on BO for iterative run‐to‐run experiments, focusing on strategies for optimizing titer values based on a mammalian process model (De Luca 2023). Their research identified that strategies incorporating constraints on the prediction interval width generally aligned model predictions more closely with experimental outcomes. The constraints were implemented as a support vector machine defining the multi‐dimensional input of the process data as a validity domain of the model, as described by Schweidtmann et al (Schweidtmann et al. 2022). Surprisingly, acquisition functions aiming at exploring the design space achieved higher titers than those aiming at maximizing the upper prediction limit of the target variable.

#### 3.1.3 Fermentation

Eskanderi et al. demonstrated the application of multi‐fidelity BO to optimize the gas conversion rate in an industrial‐scale bioreactor for syngas fermentation using a high‐fidelity computational fluid dynamics model leveraged with a simpler, ideal‐mixing‐based model (Eskandari et al. 2023). Furthermore, Thompson et al. developed a physically constrained neural network and combined it with an expected information gain acquisition function to optimize the operating conditions of a bioreactor for microbial communities (Thompson et al. 2023).

#### 3.1.4 Reaction Optimization

Rosa et al. optimized a set of 12 reaction parameters to maximize the mRNA concentration by an in vitro transcription reaction (Rosa 2022). The authors could optimize the reaction by applying a batch optimization technique of three to five sampling points, resulting in a final mRNA concentration of 12 g/L, constituting a two‐fold increase in half of the time in contrast to published industrial standards. A total of 60 experiments were performed, with 16 initial experiments in a Latin Hypercube design. Furthermore, the authors addressed a fundamental limitation GP relative to DoE: their interpretability. This was achieved by employing an explanation model based on Shapley values, which provided the authors with deeper insights and more transparent explanations.

In their study on optimizing alkaline wood delignification, Rummukainen et al. directly compared DoE and BO. They found that the choice of an initial experiment can significantly affect the BO algorithm in identifying optimal conditions quickly. Furthermore, an increasing measurement noise also slowed its progress. Bayesian optimization still provided a more precise model near the optimum when compared to a response surface model, however, in their study, the overall number of required experiments could not be reduced compared to DoE. The authors suggest combining DoE and BO: using space‐filling designs and optimal designs can help selecting initial experiments that are further refined by BO (Rummukainen 2024).

### 3.2 Downstream Processing

#### 3.2.1 Formulation

Narayanan et al. optimized three different variants of a tandem single‐chain variable fragment (scFv) derived from the antibody Humira, considering eight compounds of a buffering system as independent variables to maximize thermal stability, or to be precise, the melting temperature *T*_m_ (Narayanan 2021). Initial 20 experiments in a Latin Hypercube experimental design were used for model building. Another two cycles were performed in batch mode until 25 experiments in total were conducted to find the optimum. Compared with different DoE designs, 64–128 experiments would have been necessary to find this optimum. After initially choosing a rather conservative range of parameters, the authors also investigated the design space in extended parameter ranges. To facilitate this, experiments already conducted were leveraged as a “prior belief” to be integrated into the BO cycle, an approach not feasible with traditional DoE. Additionally, a multiple‐objective approach was performed to maximize the melting temperature and the interface stability of one scFv variant. With 15 initial experiments, a total of 33 experiments were conducted to identify the Pareto front.

Sano et al. applied BO to pharmaceutical product development to refine the formulation and manufacturing methods for orally disintegrating tablets (Sano et al. 2020). Utilizing an already published data set enhanced by data augmentation from a DoE by an artificial neural network simulation, the study sought to minimize unnecessary experiments and hasten method development. Instead of approximating the Pareto front, multiple objectives were optimized by introducing a mathematically defined score as a trade‐off of the two targets, tensile strength, and disintegration time. The findings of the study demonstrated that BO could significantly reduce the experimental workload, cutting down the number of necessary experiments from about 25, as required by the central composite DoE design, to just 10. This efficiency gain is also attributed to repeated hyperparameter tuning within the BO process, which helped to stabilize performance variations across different optimization scenarios and improve the overall average performance. Their research underscores BO's potential to lessen dependence on individual expertise in pharmaceutical development, enhancing both the efficiency and effectiveness of optimization tasks.

## 4 BO Today and Tomorrow: Tools, Techniques, and Trajectories

### 4.1 Strengths and Limitations of BO Compared to DoE

Performing a literature search in the database Scopus with the terms “Bioprocess” and “Design of Experiments” shows a steady increase of publications from 5 in the year 2009 up to 61 in the year 2024 (Kasemiire 2021). For an analogous search using only the keywords “Bayesian optimization” and “Bioprocess,” a total number of 22 can be found. Considering only those articles actually applying BO to guide experiments within a bioprocess optimization context leaves even less. This seems somewhat surprising, as most studies that compared it with the industry standard DoE report a need for less experiments and better results.

However, several factors may explain the slower adoption of BO compared to DoE. Unlike DoE, which relies on established statistical methodologies and is widely applied in industry and academia, BO demands a deeper understanding of probabilistic models, machine learning concepts, and hyperparameter tuning. This complexity might represent a barrier for experimentalists without prior experience in these areas. In addition, DoE offers simplicity and interpretability, which makes it especially valuable in regulatory settings. Its deterministic nature and fixed experimental designs are straightforward to communicate and validate, particularly in industries where regulatory approval requires transparency. BO, while offering greater flexibility and adaptability, usually relies on black box models which are harder to interpret and validate for such purposes. Another important difference is their suitability for different experimental systems. BO is suitable for nonlinear problems and scenarios with noisy or complex design spaces. For smaller‐scale, well‐characterized systems with relatively simpler objectives, DoE may provide comparable results while being easier to implement. However, BO has the advantage of leveraging prior knowledge by incorporating existing data directly into its probabilistic models. While DoE can also include sequential designs, it typically relies on predefined assumptions and model forms, limiting its ability to adapt dynamically to new insights. Finally, practical considerations, such as the availability of software tools may also influence the choice.

### 4.2 Software

DoE is implemented very well in various commercial software suites, providing good documentation and easy‐to‐use generic user interfaces. This is not the case for BO yet. Here, implementations are mainly found in open‐source software libraries for different programming languages like Python, Matlab, R, or Julia, targeted primarily for research purposes. Table 1 illustrates the variety of BO packages used in our referenced examples. Being a very active research area poses another challenge for experimentalists: no single platform currently contains all recent advancements in the field. As a result, Wang and Dowling recommend that experimentalists should not commit to a single BO software solution without first comparing the features, documentation, and tutorials of several platforms (Wang and Dowling 2022). An overview of different software packages and code examples have been given in literature multiple times and can be found in the following reviews (Jin and Kumar 2023; Zhang 2021; Wang and Dowling 2022). However, it is worth mentioning some very recently published packages which are targeted for real‐world experimental campaigns, lowering the barrier for experimentalists significantly and supporting a lot of functionalities out of the box. Some of them are developed by industrial manufacturers like BayBE, developed by Merck KGaA (Fitzner 2024), Obsidian developed by Merck & Co. Inc. (Stone and Xu 2024), or ProcessOptimizer developed by Novo Nordisk (Obdrup 2024). Occasionally, also industry software providers seem to add BO to their product portfolio (AI Experimentation at Benchling 2024; Bayesian Bioprocess Data Analytics in R&D and GMP manufacturing 2024; Desice 2024). The emergence of these tools highlights the growing interest in making BO accessible to a wider audience. The authors expect to see more software solutions in the future and therefore suspect the application of BO will increase.

**Table 1:** Overview of published applications of BO in bioprocess engineering.

Process/application	Methodology	Surrogate model	Software	Reference	Year
Media development	Multi‐information BO	Gaussian process	BoTorch	(Cosenza et al. (2022))	2022
Media development	Multi‐objective multi‐information BO	Gaussian process	BoTorch	(Cosenza (2023))	2023
Media development	BO	DNN‐BO	GPyOpt	(Yoshida et al. (2023))	2023
High‐throughput media development	Batch BO	Gaussian process	GPyOpt	(Genki and Kanda (2022))	2022
High‐throughput media development	Batch BO	Gaussian process	KriKit (MATLAB)	(Morschett (2017))	2017
High‐throughput protein engineering	Batch BO	Bayesian statistical model	PyMC, pyrff	(Helleckes et al. (2024))	2024
Cell culture /Purification	Noisy batch BO	Gaussian process	BoTorch	(Claes et al. (2024))	2024
Cell culture	BO	Gaussian process	PyBO	(Mehrian et al. (2018))	2018
Cell culture	Multiple‐objective batch BO	Gaussian process	MOBOpt, modified	(Bader (2023))	2023
Cell culture	Batch BO	Hybrid Gaussian process	MATLAB	(De Luca (2023))	2023
Fermentation	Multiple‐objective high‐dimensional BO	Gaussian process	n/a	(Liang and Lai (2021))	2021
Fermentation	BO	Physically constrained Recurrent Neural Network	n/a	(Thompson et al. (2023))	2023
Fermentation	Multi‐fidelity BO	Gaussian process	BoTorch	(Eskandari et al. (2023))	2023
High‐throughput fermentation	Batch BO	Gaussian process	MATLAB	(Freier (2016))	2016
High‐throughput fermentation	Batch BO	Bayesian statistical model	PyMC, pyrff	(Helleckes et al. (2023))	2023
Reaction engineering	BO	Gaussian process	BoTorch	(Rummukainen (2024))	2024
Reaction engineering	BO	Gaussian process	scikit‐optimize	(Rosa (2022))	2022
Reaction engineering	Batch BO	Gaussian process	PyMC	(Siedentop (2024))	2024
Reaction engineering	Human‐in‐the‐loop BO	Gaussian Process	gpjax	(Savage and Chanona (2024))	2024
Chromatography	BO	Gaussian process	scikit‐optimize, modified	(Jäpel and Buyel (2022))	2022
Chromatography	Multiple‐objective BO	Gaussian process	KriKit (MATLAB)	(Freier and von Lieres (2017))	2017
Formulation	BO	Gaussian process	COMBO	(Sano et al. (2020))	2020
Formulation	Batch BO, Multiple‐objective Batch BO	Gaussian process	scikit‐optimize, GPflowOpt	(Narayanan (2021))	2021

### 4.3 Towards Autonomous Experimentation With Bayesian

#### 4.3.1 Optimization

BO has also gained a lot of attention in the context of autonomous experimentation. Due to its cyclic character and integrated decision‐making intelligence, robots can be equipped with an algorithm that enables them to independently guide experiments, once experiments are planned. A noteworthy example from chemistry showed the optimization of a photocatalyst for hydrogen production by a robot that operated for 8 days autonomously performing nearly 700 experiments to explore a ten‐dimension design space (Burger 2020). In bioprocess engineering, multiple examples of high‐throughput screening combined with model‐aided decision‐making have been demonstrated: Kanda et al. developed an autonomous robotic AI system equipped with a batch Bayesian optimization algorithm (Genki and Kanda 2022). This system was tasked with optimizing the induction of differentiation from induced pluripotent stem cells (iPSCs) to retinal pigment epithelial (RPE) cells. It tested 143 different conditions over 111 days, navigating through 200 million potential experimental setups. The results showed an 88% improvement in iPSCRPE cell production in terms of pigmentation scores compared to the best manual cultures previously established. Helleckes et al. focused on improving the secretion of PETase enzymes by *Corynebacterium glutamicum* for the biodegradation of polyethylene terephthalate (PET) plastics (Helleckes et al. 2023). They introduced a probability based screening strategy that combines laboratory automation with BO. This approach enables rapid and efficient identification of optimal signal peptides for enzyme secretion through high‐throughput screening. By integrating automated processes with statistical modeling, the study significantly enhances the screening efficiency, identifying the most promising signal peptides for PETase secretion in just two rounds of screening. Similarly, Helleckes et al. recently developed a semi‐automated process for creating 63 different catalytically active inclusion body variants, coupled with an automated screening workflow that was optimized using Bayesian optimization and Thompson sampling (Helleckes et al. 2024).

In combination with screening methods, Bayesian optimization offers a promising way to efficiently guide through vast design spaces that were also not accessible by rigid screening alone.

### 4.4 Physics‐Informed Bayesian Optimization

Originally developed as a method for black‐box optimization, Bayesian optimization traditionally relies on data‐driven surrogate models to approximate the objective function. In recent years, the intersection of machine learning with first‐principle models has attracted significant interest across various scientific and engineering disciplines (Rackauckas 2021). This approach uses the predictive power of data‐driven methods combined with the fundamental insights offered by physical knowledge, aiming to enhance predictive accuracy to reduce computational costs and improve model interpretability. Known as hybrid modeling, this practice has a long history within the bioprocess engineering field going back to the 1990s (Psichogios and Ungar 1992). Therefore, the fusion of hybrid surrogate models with acquisition functions that exploit uncertainty quantification presents an attractive approach that could gain interest. While not widely explored in the literature, some studies within materials science and physics report that this approach leads to improved accuracy and faster identification of the global optimum compared to traditional black‐box methods (Hanuka et al. 2021; Oikonomou 2023).

The flexibility of BO offers multiple possibilities for integrating physical knowledge into the optimization process. For example, Di Fiore et al. propose a physics‐aware multi‐fidelity BO framework that initially uses low‐fidelity models based on first principles for broad exploration. This process identifies key areas where high‐fidelity experimental data or simulations are subsequently applied to refine the model (Di Fiore and Mainini 2024). However, most approaches target the model structure itself. An example demonstrates Ziatdinov et al. who developed an augmented Gaussian process‐based BO method that enhances optimization by incorporating physical models as structured probabilistic priors. This integration improves both the efficiency and accuracy of the learning process (Ziatdinov et al. 2022). Similarly, Khatamsaz et al. showed a Physics‐Informed BO enhancing optimization efficiency by incorporating physics‐infused kernels into the Gaussian process model, effectively leveraging both statistical and physical information for material design optimization (Khatamsaz 2023).

## 5 Conclusion

BO has positioned itself as a very promising process development tool in the field of bioprocess engineering, offering a sample‐efficient and adaptive optimization tool that combines machine learning and experimental decision‐making. This review article highlights its key principles and showcases different currently published applications across different unit operations in bioprocess engineering. The software landscape as well as applications in autonomous experimentation are discussed. Additionally, the exploration of combining BO with physics‐based models is presented, indicating a promising direction for integrating data‐driven insights with established scientific principles.

## Author Contributions

Florian Gisperg and Oliver Spadiut designed the study. Florian Gisperg performed the literature research of this study. Robert Klausser, Mohamed Elshazly, Julian Kopp, and Eva Přáda Brichtová gave valuable scientific input regarding the paper draft. Florian Gisperg drafted the manuscript. Robert Klausser, Mohamed Elshazly, Julian Kopp, Eva Přáda Brichtová and Oliver Spadiut reviewed the manuscript.

## Conflicts of Interest

The authors declare that the research was conducted in the absence of any commercial or financial relationships that could be construed as a potential conflicts of interest.

## Data Availability

The data that support the findings of this study are available on request from the corresponding author. The authors have nothing to report.

## References

[bit28960-bib-0001] AI Experimentation at Benchling en. April. https://www.benchling.com/ai. 2024

[bit28960-bib-0002] Bader, J. , H. Narayanan , P. Arosio , and J. C. Leroux . 2023. “Improving Extracellular Vesicles Production Through a Bayesian Optimization‐Based Experimental Design.”European Journal of Pharmaceutics and Biopharmaceutics 182 January: 103–114. 10.1016/j.ejpb.2022.12.004. 36526027

[bit28960-bib-0003] Balandat, Maximilian , et al. “BOTORCH: A Framework for Efficient Montecarlo Bayesian Optimization.” In: Proceedings of the 34th International Conference on Neural Information Processing Systems. NIPS’20. Red Hook, NY, USA: Curran Associates Inc., 2020, pp. 21524–21538.

[bit28960-bib-0004] Bayes, Thomas . 1763. “Lii. An Essay Towards Solving a Problem in the Doctrine of Chances. by the Late Rev. Mr. Bayes, F. R. S. Communicated by Mr. Price, in a Letter to John Canton, A. M. F. R. S.”Philosophical Transactions of the Royal Society of London 53 December: 370–418. 10.1098/rstl.1763.0053.

[bit28960-bib-0005] Bayesian Bioprocess Data Analytics in R&D and GMP manufacturing . enUS. April. https://www.koerber‐pharma.com/en/blog/bayesian‐bioprocess‐data‐analyticsin‐rd‐and‐gmp‐manufacturing. 2024

[bit28960-bib-0006] Box, G. E. P. , and K. B. Wilson . 1951). Publisher: [Royal Statistical Society, Wiley]. “On the Experimental Attainment of Optimum Conditions.” Journal of the Royal Statistical Society Series B: Statistical Methodology 13, no. 1: 1–38. https://www.jstor.org/stable/2983966.

[bit28960-bib-0007] Bradford, Eric , Artur M. Schweidtmann , and Alexei Lapkin . 2018. “Efficient Multiobjective Optimization Employing Gaussian Processes, Spectral Sampling and a Genetic Algorithm.” Journal of Global Optimization 71, no. 2 June: 407–438. 10.1007/s10898-018-06092.

[bit28960-bib-0008] Burger, B. , P. M. Maffettone , V. V. Gusev , et al. 2020). Publisher: Nature Publishing Group. “A Mobile Robotic Chemist.”Nature 583, no. 7815 July: 237–241. 10.1038/s41586-020-2442-2. 32641813

[bit28960-bib-0009] Chepiga, T. , P. Zhilyaev , A. Ryabov , et al. 2023). Number: 3 Publisher: Multidisciplinary Digital Publishing Institute. “Process Parameter Selection for Production of Stainless Steel 316L Using Efficient Multi‐Objective Bayesian Optimization Algorithm.”Materials 16, no. 3 January: 1050. 10.3390/ma16031050. 36770057 PMC9919176

[bit28960-bib-0010] Choinière, L. , Guay‐Hottin, R. , Picard, R. , Lajoie, G. , Bonizzato, M. , & Dancause, N. (2024). Gaussian‐process‐based Bayesian optimization for neurostimulation interventions in rats. STAR Protocols 5 no. 1: 102885. 10.1016/j.xpro.2024.102885. 38358881 PMC10876592

[bit28960-bib-0011] Claes, E. , T. Heck , K. Coddens , M. Sonnaert , J. Schrooten , and J. Verwaeren . 2024. “Bayesian Cell Therapy Process Optimization.”Biotechnology and Bioengineering 121: 1569–1582. 10.1002/bit.28669. 38372656

[bit28960-bib-0012] Cosenza, Z. , R. Astudillo , P. I. Frazier , K. Baar , and D. E. Block . 2022. “Multi‐Information Source Bayesian Optimization of Culture Media for Cellular Agriculture.”Biotechnology and Bioengineering 119, no. 9: 2447–2458. 10.1002/bit.28132. 35538846 PMC9541924

[bit28960-bib-0013] Cosenza, Z. , D. E. Block , and K. Baar . 2021. “Optimization of Muscle Cell Culture Media Using Nonlinear Design of Experiments.” Biotechnology Journal 16, no. 11: 2100228. 10.1002/biot.202100228. 34387397

[bit28960-bib-0014] Cosenza, Z. , D. E. Block , K. Baar , and X. Chen 2023. “Multi‐Objective Bayesian Algorithm Automatically Discovers Low‐Cost High‐Growth Serum‐Free Media for Cellular Agriculture Application.”Engineering in Life Sciences 23, no. 8: e2300005. 10.1002/elsc.202300005. 37533728 PMC10390662

[bit28960-bib-0015] Csató, L. , and M. Opper . 2002. “Sparse On‐Line Gaussian Processes.”Neural Computation 14, no. 3 March: 641–668. 10.1162/089976602317250933. 11860686

[bit28960-bib-0016] Daniel, C. 1973. “One‐At‐A‐Time Plans.” Journal of the American Statistical Association 68, no. 342 June: 353–360. 10.1080/01621459.1973.10482433.

[bit28960-bib-0017] Daniel Golovin , et al. “Google Vizier: A Service for Black‐Box Optimization.”In: Proceedings of the 23rd ACM SIGKDD International Conference on Knowledge Discovery and Data Mining. Halifax NS Canada: ACM, August 2017, pp. 1487–1495. 10.1145/3097983.3098043.

[bit28960-bib-0018] Desice . ‐ Design of Experiments (DoE). 2024. https://www.desice.io/.

[bit28960-bib-0019] De Luca, R. , G. Costa , H. Narayanan , et al. 2023. “Comparison of Strategies for Iterative Model‐Based Upstream Bioprocess Development With Single and Parallel Reactor Set‐Ups.” en.” Biochemical Engineering Journal 191 February: 108813. 10.1016/j.bej.2023.108813.

[bit28960-bib-0020] Di Fiore, F. , and L. Mainini . 2024. “Physics‐Aware Multifidelity Bayesian Optimization: A Generalized Formulation.”Computers & Structures 296: 107302. 10.1016/j.compstruc.2024.107302.

[bit28960-bib-0021] Eskandari, Mahdi , Lars Puiman , and Jakob Zeitler . Multi‐fidelity Bayesian Optimisation of Syngas Fermentation Simulators . arXiv:2311.05776 [cs, eess]. November 2023. http://arxiv.org/abs/2311.05776.

[bit28960-bib-0022] Fisher, R. A. 1935. isbn: 978‐0‐02‐844690‐5. The Design of Experiments (eng. 1. ed. OCLC: 471778573). Hafner Press.

[bit28960-bib-0023] Fitzner, Martin , et al. BayBE — A Bayesian Back End for Design of Experiments . original‐date: 2023‐11‐27T17:02:40Z. Darmstadt, Germany, March 2024. https://github.com/emdgroup/baybe.

[bit28960-bib-0024] Frazier, Peter I. A Tutorial on Bayesian Optimization. arXiv:1807.02811 [cs, math, stat]. July 2018. 10.48550/arXiv.1807.02811.

[bit28960-bib-0025] Frazier, Peter I. , Warren B. Powell , and Savas Dayanik . “A Knowledge Gradient Policy for Sequential Information Collection.”(US). In: SIAM Journal on Control and Optimization 47.5 (2008). Publisher: Society for Industrial and Applied Mathematics Publications, pp. 2410–2439. 10.1137/070693424.

[bit28960-bib-0026] Frazier, Peter I. , and Jialei Wang . 2015. “Bayesian Optimization for Materials Design.” In Springer Series in Materials Science, 45–75. Springer International Publishing, December. 10.1007/978-3-319-23871-5_3.

[bit28960-bib-0027] Freier, L. , and E. von Lieres . 2017. “Multi‐Objective Global Optimization (MOGO): Algorithm and Case Study in Gradient Elution Chromatography.” en.” Biotechnology Journal 12, no. 7: 1600613. https://onlinelibrary.wiley.com/doi/pdf/10.1002/b. 10.1002/biot.201600613 28008726

[bit28960-bib-0028] Freier, L. , J. Hemmerich , K. Schöler , W. Wiechert , M. Oldiges , and E. von Lieres . 2016). eprint:. “Framework for Kriging‐Based Iterative Experimental Analysis and Design: Optimization of Secretory Protein Production in Corynebacterium Glutamicum.” en.” Engineering in Life Sciences 16, no. 6: 538–549. issn: 1618‐2863. 10.1002/elsc.201500171.

[bit28960-bib-0029] Garnett, Roman . 2023. Bayesian Optimization. Cambridge University Press.

[bit28960-bib-0030] Genki, N. , Kanda , et al. “Robotic Search for Optimal Cell Culture in Regenerative Medicine.” In: eLife 11 (June 2022). Ed. by Simón Méndez‐Ferrer , et al. Publisher: eLife Sciences Publications, Ltd, e77007. 10.7554/eLife.77007. PMC9239686 35762203

[bit28960-bib-0031] Gonzalez, Javier , et al. “Batch Bayesian Optimization via Local Penalization.” en. In: (2015). 10.48550/arXiv.1505.08052.

[bit28960-bib-0032] Goos, Peter . 2006). eprint. “Optimal Versus Orthogonal and Equivalent‐Estimation Design of Blocked and Split‐Plot Experiments.” en.” Statistica Neerlandica 60, no. 3: 361–378. 10.1111/j.1467-9574.2006.00333.x.

[bit28960-bib-0033] Greenhill, S. , S. Rana , S. Gupta , P. Vellanki , and S. Venkatesh . 2020). Conference Name: IEEE. Access. “Bayesian Optimization for Adaptive Experimental Design: A Review.” IEEE Access 8: 13937–13948. 10.1109/ACCESS.2020.2966228.

[bit28960-bib-0034] Hanuka, A. , X. Huang , J. Shtalenkova , et al. 2021. “Physics Model‐Informed Gaussian Process for Online Optimization of Particle Accelerators.” en.” Physical Review Accelerators and Beams 24, no. 7 July: 072802. 10.1103/PhysRevAccelBeams.24.072802.

[bit28960-bib-0035] Helleckes, L. M. , K. Küsters , C. Wagner , et al. 2024. “High‐Throughput Screening of Catalytically Active Inclusion Bodies Using Laboratory Automation and Bayesian Optimization.” eng”.” Microbial Cell Factories 23, no. 1 February: 67. 10.1186/s12934-024-02319-y. 38402403 PMC10894497

[bit28960-bib-0036] Helleckes, L. M. , C. Müller , T. Griesbach , et al. 2023. “Explore or Exploit? A Model‐Based Screening Strategy for Petase Secretion by Corynebacterium Glutamicum.” English.” Biotechnology and Bioengineering 120, no. 1: 139–153. 10.1002/bit.28261. 36225165

[bit28960-bib-0037] Hu, R. , L. Fu , Y. Chen , J. Chen , Y. Qiao , and T. Si . 2023. “Protein Engineering Via Bayesian Optimization‐Guided Evolutionary Algorithm and Robotic Experiments.” eng.” Briefings in Bioinformatics 24, no. 1 January: bbac570. 10.1093/bib/bbac570. 36562723

[bit28960-bib-0038] Jäpel, R. C. , and J. F. Buyel . 2022. “Bayesian Optimization Using Multiple Directional Objective Functions Allows the Rapid Inverse Fitting of Parameters for Chromatography Simulations.” English.” Journal of Chromatography A 1679: 463408. 10.1016/j.chroma.2022.463408. 35977456

[bit28960-bib-0039] Jin, Y. , and P. V. Kumar . 2023. “Bayesian Optimisation for Efficient Material Discovery: A Mini Review.” English.” Nanoscale 15, no. 26: 10975–10984. 10.1039/d2nr07147a. 37337888

[bit28960-bib-0040] Theckel Joy, T. , S. Rana , S. Gupta , and S. Venkatesh . 2019. “A Flexible Transfer Learning Framework for Bayesian Optimization With Convergence Guarantee.” Expert Systems with Applications 115 January: 656–672. 10.1016/j.eswa.2018.08.023.

[bit28960-bib-0041] Kasemiire, A. , H. T. Avohou , C. De Bleye , et al. 2021. “Design of Experiments and Design Space Approaches in the Pharmaceutical Bioprocess Optimization.” European Journal of Pharmaceutics and Biopharmaceutics 166 September: 144–154. 10.1016/j.ejpb.2021.06.004. 34147574

[bit28960-bib-0042] Khatamsaz, D. , R. Neuberger , A. M. Roy , S. H. Zadeh , R. Otis , and R. Arróyave . 2023). Number: 1 Publisher: Nature Publishing Group. “A Physics Informed Bayesian Optimization Approach for Material Design: Application to Niti Shape Memory Alloys.” en.” npj Computational Materials 9, no. 1 December: 1–11. 10.1038/s41524-023-01173-7.

[bit28960-bib-0043] Kushner, H. J. 1964. “A New Method of Locating the Maximum Point of an Arbitrary Multipeak Curve in the Presence of Noise.” Journal of Basic Engineering 86: 97–106. https://api.semanticscholar.org/CorpusID:62599010.

[bit28960-bib-0044] Letham, B. , B. Karrer , G. Ottoni , and E. Bakshy . 2019). Publisher: International Society for Bayesian Analysis. “Constrained Bayesian Optimization with Noisy Experiments.” Bayesian Analysis 14, no. 2 June: 495–519. 10.1214/18-BA1110.

[bit28960-bib-0045] Liang, Qiaohao , and Lipeng Lai . “Scalable Bayesian Optimization Accelerates Process Optimization of Penicillin Production. en.” In:. 2021. https://openreview.net/forum?id=UVdSYXMNdOe. Oct.

[bit28960-bib-0046] Močkus, J. 1975. “On Bayesian Methods for Seeking the Extremum.” In en.” Optimization Techniques IFIP Technical Conference Novosibirsk, July 1–7, 1974, edited by G. I. Marchuk , 400–404.: Springer. 10.1007/3-540-07165-2_55.

[bit28960-bib-0047] Mehrian, M. , Y. Guyot , I. Papantoniou , et al. 2018. “Maximizing Neotissue Growth Kinetics in a Perfusion Bioreactor: An in Silico Strategy Using Model Reduction and Bayesian Optimization.” eng.” Biotechnology and Bioengineering 115, no. 3 March: 617–629. 10.1002/bit.26500. 29205280

[bit28960-bib-0048] Morschett, H. , L. Freier , J. Rohde , W. Wiechert , E. von Lieres , and M. Oldiges . 2017. “A Framework for Accelerated Phototrophic Bioprocess Development: Integration of Parallelized Microscale Cultivation, Laboratory Automation and Kriging‐Assisted Experimental Design.” Biotechnology for Biofuels 10, no. 1 January: 26. 10.1186/s13068-017-0711-6. 28163783 PMC5282810

[bit28960-bib-0049] Narayanan, H. , F. Dingfelder , I. Condado Morales , et al. 2021. Publisher: American Chemical Society. “Design of Biopharmaceutical Formulations Accelerated by Machine Learning.” Molecular Pharmaceutics 18, no. 10 October: 3843–3853. 10.1021/acs.molpharmaceut.1c00469. 34519511

[bit28960-bib-0050] Nguyen, Quan , Luis Serrano , and David Sweet . 2023. Bayesian Optimization in Action. eng. Manning.

[bit28960-bib-0051] Obdrup, Aksel , et al. ProcessOptimizer . November 2024. 10.5281/zenodo.14179617: https://zenodo.org/records/14179617.

[bit28960-bib-0052] Oikonomou, A. , T. Loutas , D. Fan , et al. 2023). Number: 1 Publisher: Nature Publishing Group. “Physics‐Informed Bayesian Learning of Electrohydrodynamic Polymer Jet Printing Dynamics.” en.” Communications Engineering 2, no. 1 April: 1–15. 10.1038/s44172-023-00069-0.

[bit28960-bib-0053] Picheny, Victor , et al. “Bayesian Optimization Under Mixed Constraints With a Slack‐Variable Augmented Lagrangian.” In: Advances in Neural Information Processing Systems. Vol. 29. Curran Associates Inc., 2016. url: https://proceedings.neurips.cc/paper_files/paper/2016/hash/31839b036f63806cba3f47b93af.

[bit28960-bib-0054] Pickles, Thomas , et al. 2024. “Comparative Study on Adaptive Bayesian Optimization for Batch Cooling Crystallization for Slow and Fast Kinetic Regimes.” Crystal Growth & Design 24, no. 3: 1245–1253. 10.1021/acs.cgd.3c01225. 38344674 PMC10853904

[bit28960-bib-0055] Psichogios, D. C. , and L. H. Ungar . 1992. MAG ID: 1968326286. “A Hybrid Neural Network‐First Principles Approach to Process Modeling.” AIChE Journal 38, no. 10 October: 1499–1511. 10.1002/aic.690381003.

[bit28960-bib-0056] Qi, C. , T. Hu , J. Zheng , et al. 2024. “Artificial Intelligence‐Based Prediction Model for the Elemental Occurrence Form of Tailings and Mine Wastes.” English.” Environmental Research 249: 118378. 10.1016/j.envres.2024.118378. 38311206

[bit28960-bib-0057] Rackauckas, Christopher , et al. Universal Differential Equations for Scientific Machine Learning. arXiv:2001.04385 [cs, math, q‐bio, stat]. November 2021. 10.48550/arXiv.2001.04385.

[bit28960-bib-0058] Rasmussen, Carl Edward , and Christopher K. I. Williams . 2006. isbn: 978‐0262‐18253‐9. Gaussian Processes for Machine Learning. en. Adaptive computation and machine learning. OCLC: ocm61285753. MIT Press.

[bit28960-bib-0059] Razavi, S. , and H. V. Gupta . 2015. “What Do We Mean by Sensitivity Analysis? The Need for Comprehensive Characterization of “Global” Sensitivity in Earth and Environmental Systems Models.” en.” Water Resources Research 51, no. 5: 3070–3092. 10.1002/2014WR016527.

[bit28960-bib-0060] Rohr, B. , H. S. Stein , D. Guevarra , et al. 2020. Publisher: The Royal Society of Chemistry. “Benchmarking the Acceleration of Materials Discovery by Sequential Learning.” en.” Chemical Science 11, no. 10 March : 2696–2706. 10.1039/C9SC05999G. 34084328 PMC8157525

[bit28960-bib-0061] Rosa, S. S. , D. Nunes , L. Antunes , D. Prazeres , M. Marques , and A. M. Azevedo . 2022. “Maximizing mRNA Vaccine Production With Bayesian Optimization.” en.” Biotechnology and Bioengineering 119, no. 11: 3127–3139. 10.1002/bit.28216. 36017534 PMC9539360

[bit28960-bib-0062] Rummukainen, H. , H. Hörhammer , P. Kuusela , J. Kilpi , J. Sirviö , and M. Mäkelä . 2024. “Traditional or Adaptive Design of Experiments? A Pilot‐Scale Comparison on Wood Delignification.” Heliyon 10, no. 2 January: e24484. 10.1016/j.heliyon.2024.e24484. 38293354 PMC10826314

[bit28960-bib-0063] Sano, S. , T. Kadowaki , K. Tsuda , and S. Kimura . 2020. “Application of Bayesian Optimization for Pharmaceutical Product Development.” English.” Journal of Pharmaceutical Innovation 15, no. 3: 333–343. 10.1007/s12247-01909382-8.

[bit28960-bib-0064] Savage, Tom , and Ehecatl Antonio del Rio Chanona . 2024. “Human‐Algorithm Collaborative Bayesian Optimization for Engineering Systems.” Computers & Chemical Engineering 189 October: 108810. 10.1016/j.compchemeng.2024.108810.

[bit28960-bib-0065] Schweidtmann, A. M. , J. M. Weber , C. Wende , L. Netze , and A. Mitsos . 2022. “Obey Validity Limits of Data‐Driven Models Through Topological Data Analysis and One‐Class Classification.” en.” Optimization and Engineering 23, no. 2 June: 855–876. 10.1007/s11081-021-09608-0.

[bit28960-bib-0066] Shahriari, Bobak , et al. “Taking the Human Out of the Loop: A Review of Bayesian Optimization.” In: Proceedings of the IEEE 104.1 (Jan. 2016). Conference Name: Proceedings of the IEEE, pp. 148–175. 10.1109/JPROC.2015.2494218.

[bit28960-bib-0067] Shields, B. J. , J. Stevens , J. Li , et al. 2021. “Bayesian Reaction Optimization as a Tool for Chemical Synthesis.” eng.” Nature 590, no. 7844 February: 89–96. 10.1038/s41586-021-03213-y. 33536653

[bit28960-bib-0068] Siedentop, Regine , et al. 2024. “Avoiding Replicates in Biocatalysis Experiments: Machine Learning for Enzyme Cascade Optimization.” ChemCatChem 17, no. 1: e202400777. 10.1002/cctc.202400777.

[bit28960-bib-0069] Snoek, Jasper , et al. Scalable Bayesian Optimization Using Deep Neural Networks. arXiv:1502.05700 [stat]. July 2015. 10.48550/arXiv.1502.05700.

[bit28960-bib-0070] Snoek, Jasper , Hugo Larochelle , and Ryan P. Adams . 2012. “Practical Bayesian Optimization of Machine Learning Algorithms.” In Advances in Neural Information Processing Systems (25). Curran Associates, Inc. 10.48550/arXiv.1206.2944.

[bit28960-bib-0071] Srinivas, N. , A. Krause , S. M. Kakade , and M. W. Seeger . 2012). Publisher: Institute of Electrical and Electronics Engineers (IEEE). “Information‐Theoretic Regret Bounds for Gaussian Process Optimization in the Bandit Setting.” IEEE Transactions on Information Theory 58, no. 5 (May: 3250–3265. 10.1109/tit.2011.2182033.

[bit28960-bib-0072] Stone, Kevin , and Yuting Xu . Obsidian. original‐date: 2024‐08‐06T15:20:24Z. December 2024. https://github.com/MSDLLCpapers/obsidian.

[bit28960-bib-0073] Szpisják‐Gulyás, N. , A. N. Al‐Tayawi , Z. H. Horváth , Z. László , S. Kertész , and C. Hodúr . 2023. “Methods for Experimental Design, Central Composite Design and the Box–Behnken Design, to Optimise Operational Parameters: A Review.” Acta Alimentaria 52, no. 4 December: 521–537. 10.1556/066.2023.00235.

[bit28960-bib-0074] Thompson, J. C. , V. M. Zavala , and O. S. Venturelli . 2023. Publisher: Public Library of Science. “Integrating a Tailored Recurrent Neural Network Wwith Bayesian Experimental Design to Optimize Microbial Community Functions.” en.” PLoS Computational Biology 19, no. 9 September: e1011436. 10.1371/journal.pcbi.1011436. 37773951 PMC10540976

[bit28960-bib-0075] Wang, Fan , and Jian Elton Chen . 2023. “Efficient Modeling of Random Fields by Using Gaussian Process Inducing‐Point Approximations.” Computers and Geotechnics 157 May: 105304. 10.1016/j.compgeo.2023.105304.

[bit28960-bib-0076] Wang, Ke , and Alexander W. Dowling . 2022. “Bayesian Optimization for Chemical Products and Functional Materials.” Current Opinion in Chemical Engineering 36 (June: 100728. 10.1016/j.coche.2021.100728.

[bit28960-bib-0077] Wang, Xilu , et al. 2023. “Recent Advances in Bayesian Optimization.” en.” ACM Computing Surveys 55, no. 13s December: 1–36. 10.1145/3582078.

[bit28960-bib-0078] Westermann, Paul , and Ralph Evins . Using Bayesian Deep Learning Approaches for Uncertainty‐Aware Building Energy Surrogate Models. arXiv:2010.03029 [cs, stat]. October 2020. 10.48550/arXiv.2010.03029.

[bit28960-bib-0079] Wynn, H. P. 1970). Publisher: Institute of Mathematical Statistics. “The Sequential Generation of D‐Optimum Experimental Designs.” Annals of Mathematical Statistics 41, no. 5 October: 1655–1664. 10.1214/aoms/1177696809.

[bit28960-bib-0080] Yoshida, K. , K. Watanabe , T. Y. Chiou , and M. Konishi . 2023. “High Throughput Optimization of Medium Composition for *Escherichia coli* Protein Expression Using Deep Learning and Bayesian Optimization.” eng.” Journal of Bioscience and Bioengineering 135, no. 2 February: 127–133. 10.1016/j.jbiosc.2022.12.004. 36586793

[bit28960-bib-0081] Zhang, Jiyizhe , et al. 2024). Publisher: The Royal Society of Chemistry. “Multi‐Objective Bayesian Optimisation Using Q‐Noisy Expected Hypervolume Improvement (qNEHVI) for the Schotten–Baumann Reaction.” en.” Reaction Chemistry & Engineering 9, no. 3 (February: 706–712. 10.1039/D3RE00502J.

[bit28960-bib-0082] Zhang, Mimi , et al. Bayesian Optimisation for Sequential Experimental Design with Applications in Additive Manufacturing . arXiv:2107.12809 [cs]. November 2021. 10.48550/arXiv.2107.12809.

[bit28960-bib-0083] Ziatdinov, Maxim A. , Ayana Ghosh , and Sergei V. Kalinin . 2022. “Physics Makes the Difference: Bayesian Optimization and Active Learning Via Augmented Gaussian Process.” en.” Machine Learning: Science and Technology 3, no. 1 March: 015003. 10.1088/2632-2153/ac4baa.

